# Supplementary material for: Error estimates for the analysis of differential expression from RNA-seq count data
Source: PeerJ. 2014 Sep 23;2:e576. doi: 10.7717/peerj.576 (PMC4179614; doi:10.7717/peerj.576)
Supplement: File S1 [file peerj-02-576-s001.pdf]

Error estimates for the analysis of differential expression from RNA-seq count data
Supplementary material

Conrad J. Burden, Sumaira E. Qureshi and Susan R. Wilson

Figures S1 to S20:

True and estimated false discovery rates (FDRs) from synthetic datasets created with 1, 5, 10 and 15% of genes differentially expressed for $n = 2$, 3, 4, 6 and 10 replicates of control and treatment data. Seven different methods were used for calculating p-values and q-values: PoissonSeq [1], QLSpline [2], DESeq and DESeq2 [3], edgeR [4] and our proposed variants Polyfit-DESeq and Polyfit-edgeR (labelled with the extension_PF). Solid curves: true FDRs. Broken curves: estimated FDRs. The true FDR curves do not differ noticeably on the scale of the plots between DESeq and Polyfit-DESeq or between edgeR and Polyfit-edgeR, so only the DESeq and edgeR true FDR curves are shown. Each right hand plot is an expanded view of the neighbouring left hand plot restricted to the subset of genes up to a significance point roughly corresponding to the number of truly DE genes.

References

[1] Li J, Witten D, Johnstone I, Tibshirani R: **Normalization, testing, and false discovery rate estimation for RNA-sequencing data**. *Biostatistics* 2012, **13**(3):523–538.

[2] Lund S, Nettleton D, McCarthy D, Smyth G: **Detecting differential expression in RNA-sequence data using quasi-likelihood with shrunken dispersion estimates**. *Statistical Applications in Genetics and Molecular Biology* 2012, **11**(5):Article 8.

[3] Anders S, Huber W: **Differential expression analysis for sequence count data**. *Genome Biology* 2010, **11**(10):R106.

[4] Robinson MD, Oshlack A: **A scaling normalization method for differential expression analysis of RNA-seq data**. *Genome Biology* 2010, **11**:R25.

[5] Bottomly et al D: **Evaluating Gene Expression in C57BL/6J and DBA/2J Mouse Striatum Using RNA-Seq and Microarrays**. *PLoS ONE* 2011, **6**:e17820.

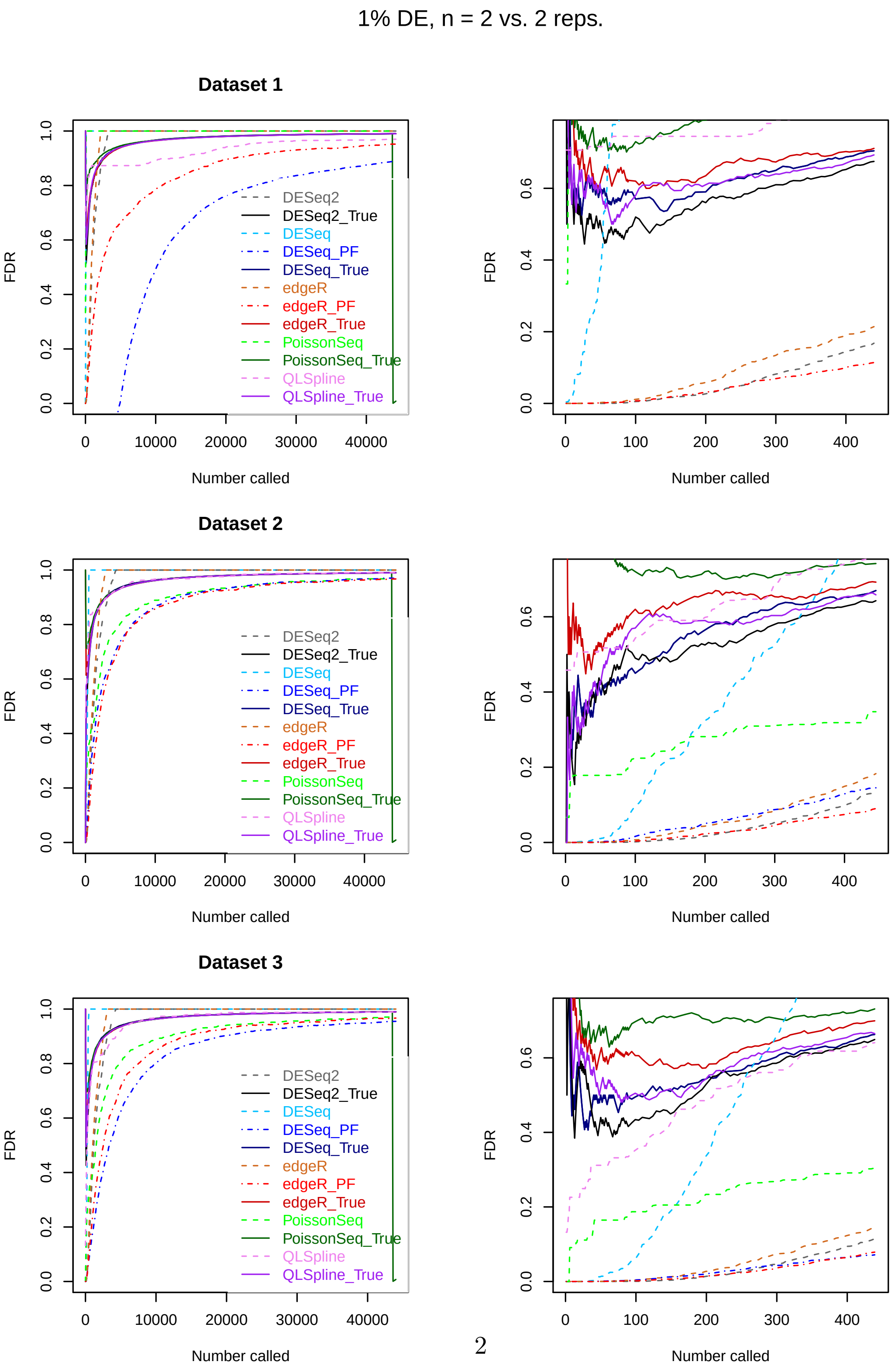

Figure S1: $n = 2$, DE $= 1\%$

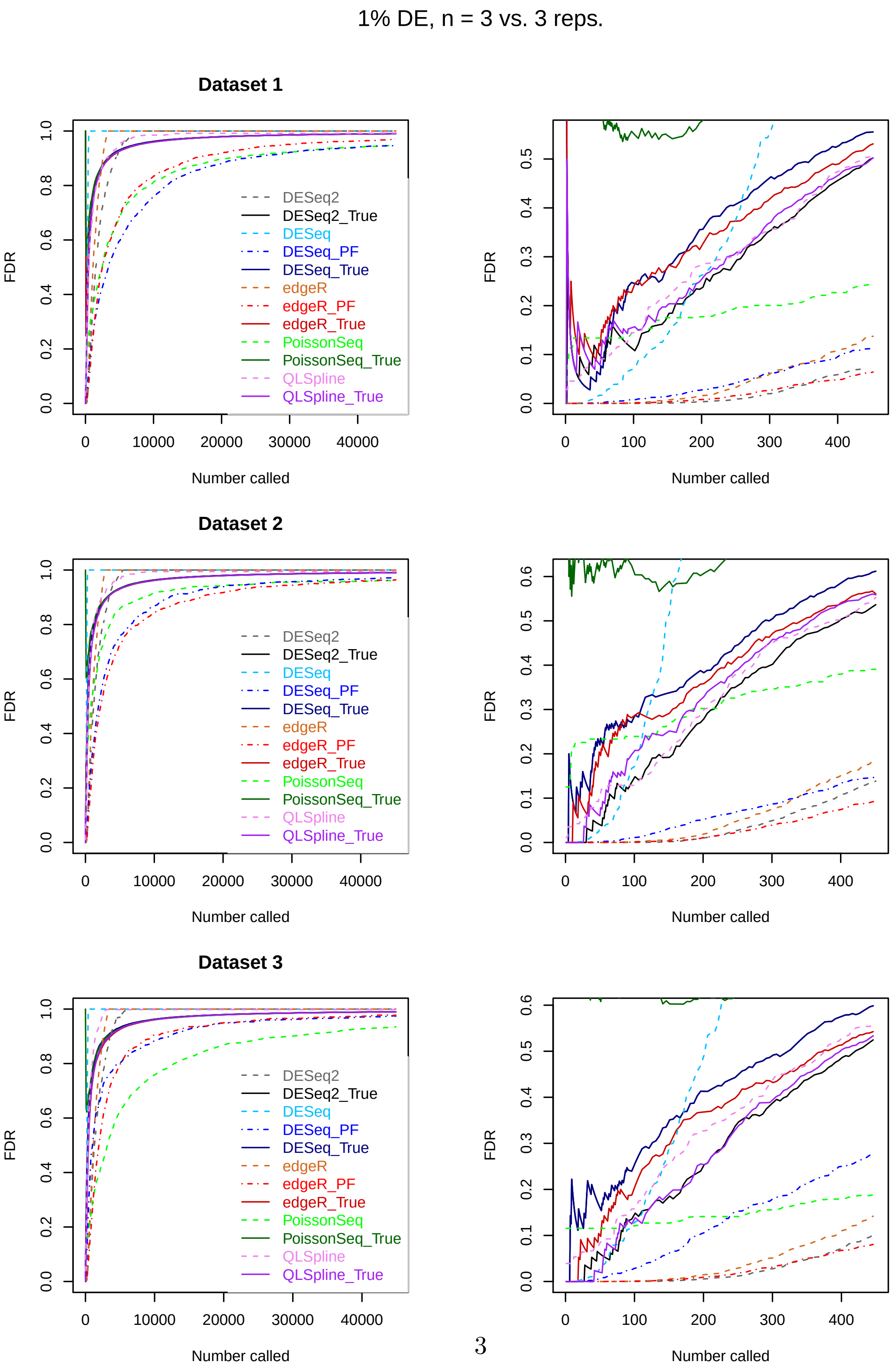

Figure S2: $n = 3$, DE $= 1\%$

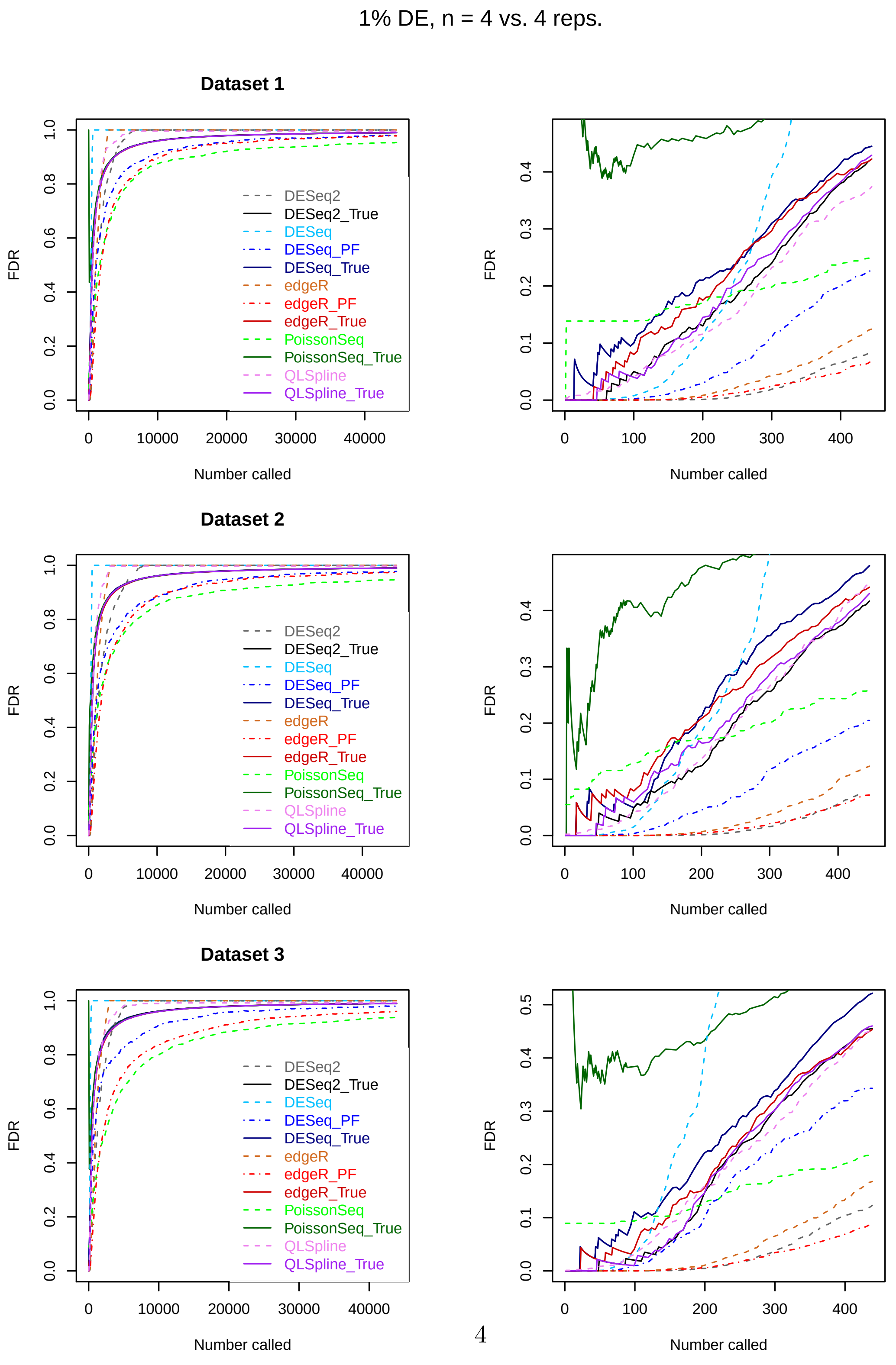

Figure S3: $n = 4$, DE $= 1\%$

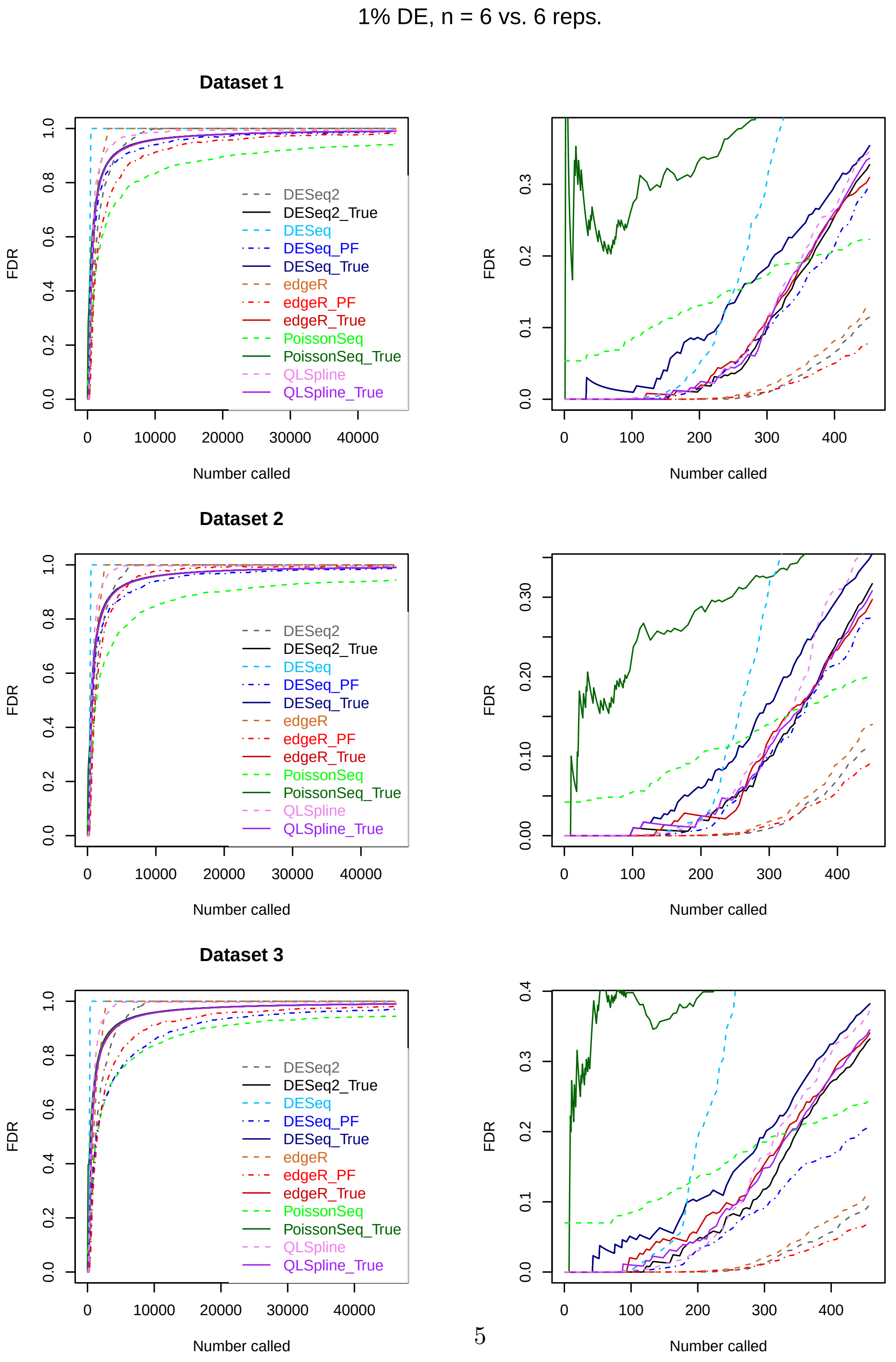

Figure S4: $n = 6$, DE $= 1\%$

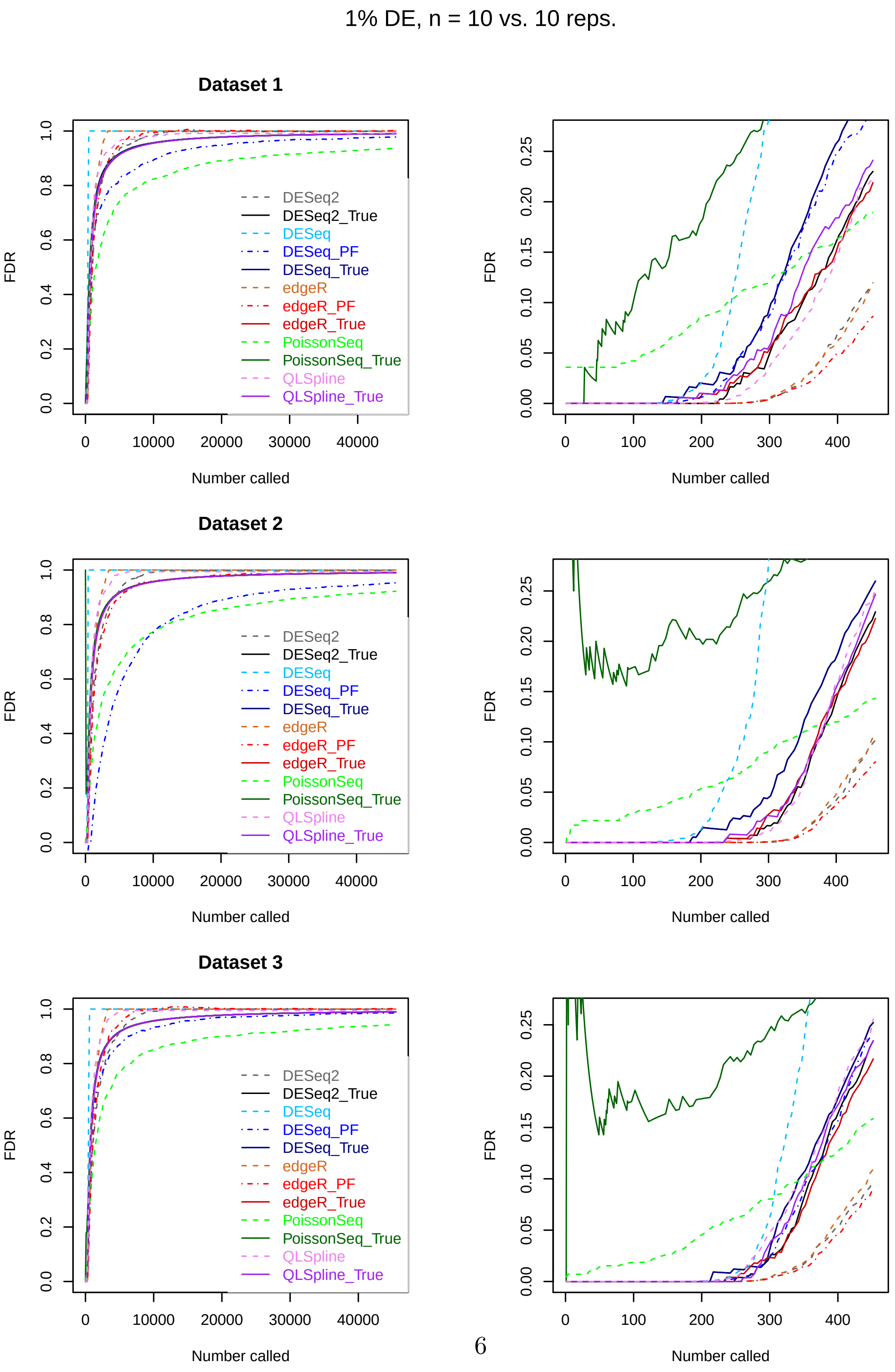

Figure S5: $n = 10$, DE $= 1\%$

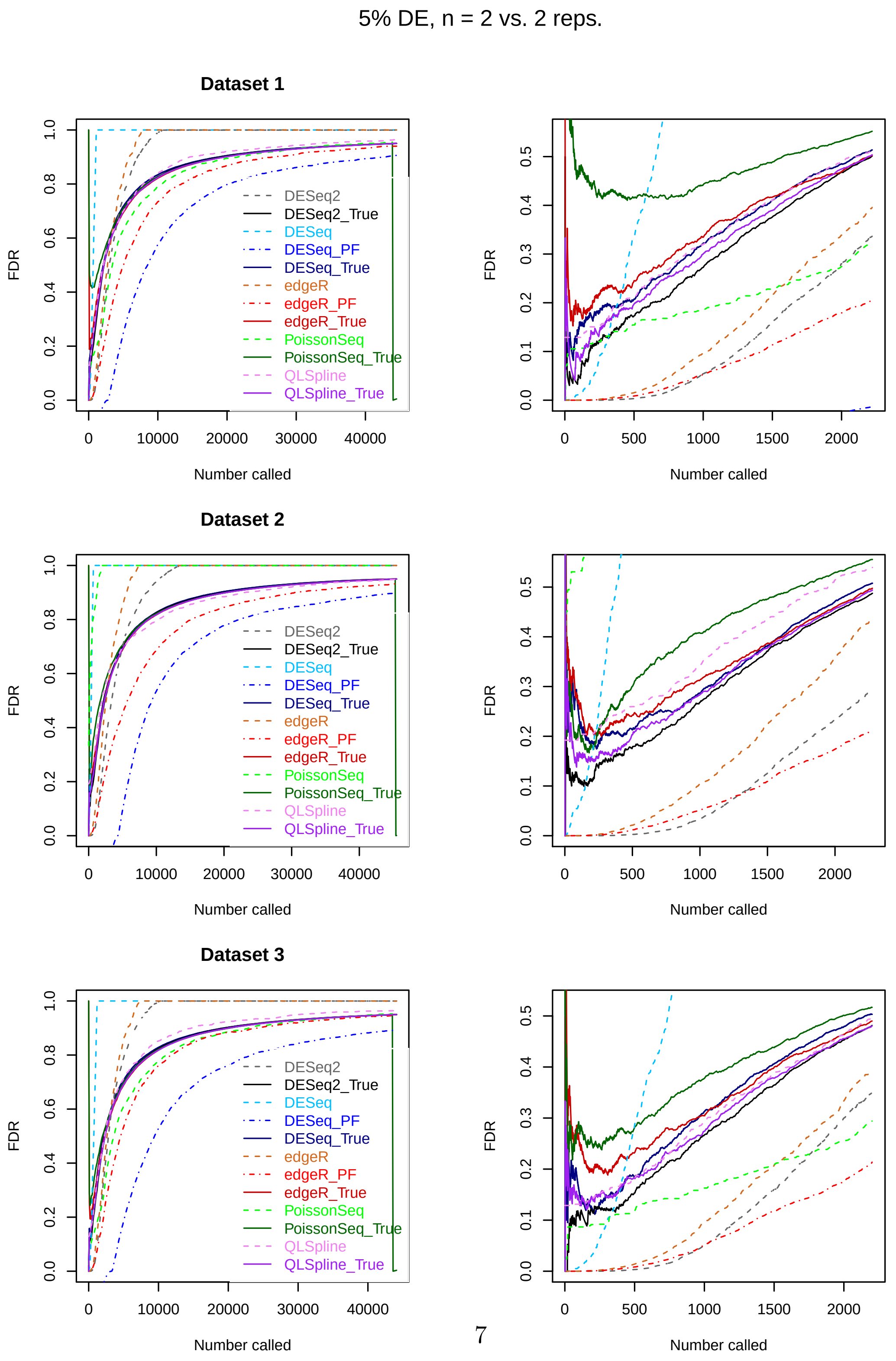

Figure S6: $n = 2$, DE $= 5\%$

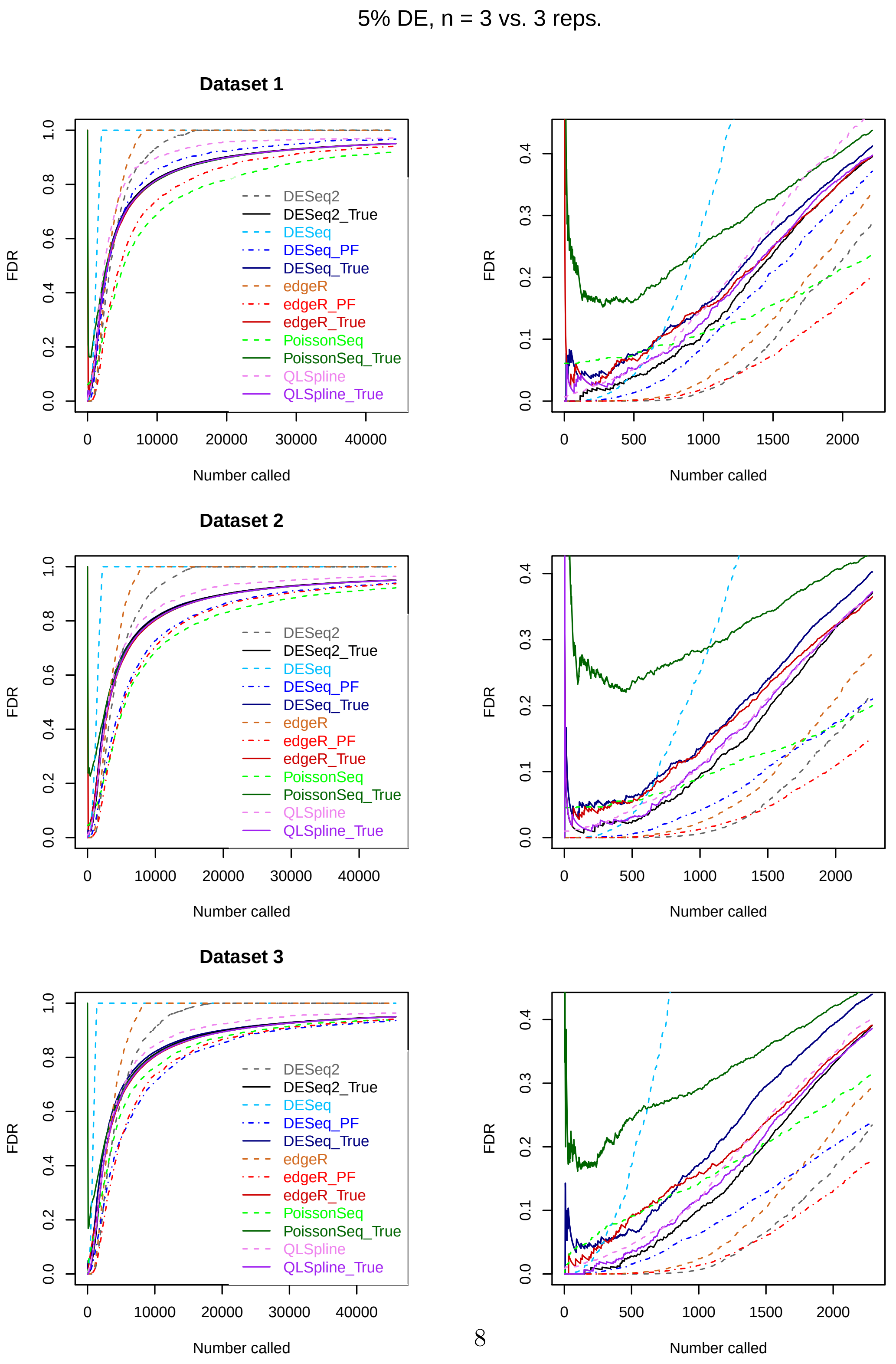

Figure S7: $n = 3$, DE $= 5\%$

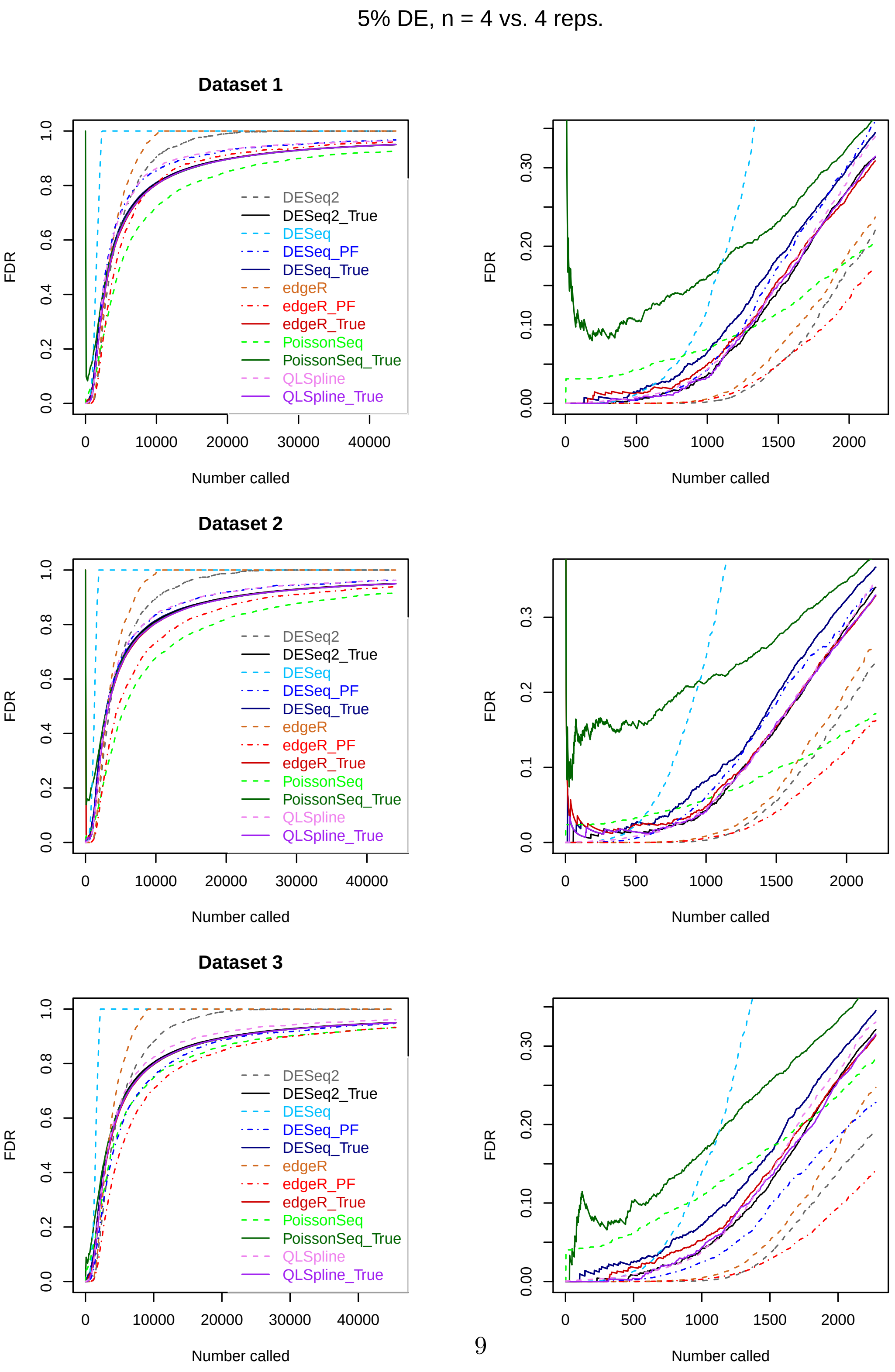

Figure S8: $n = 4$, DE $= 5\%$

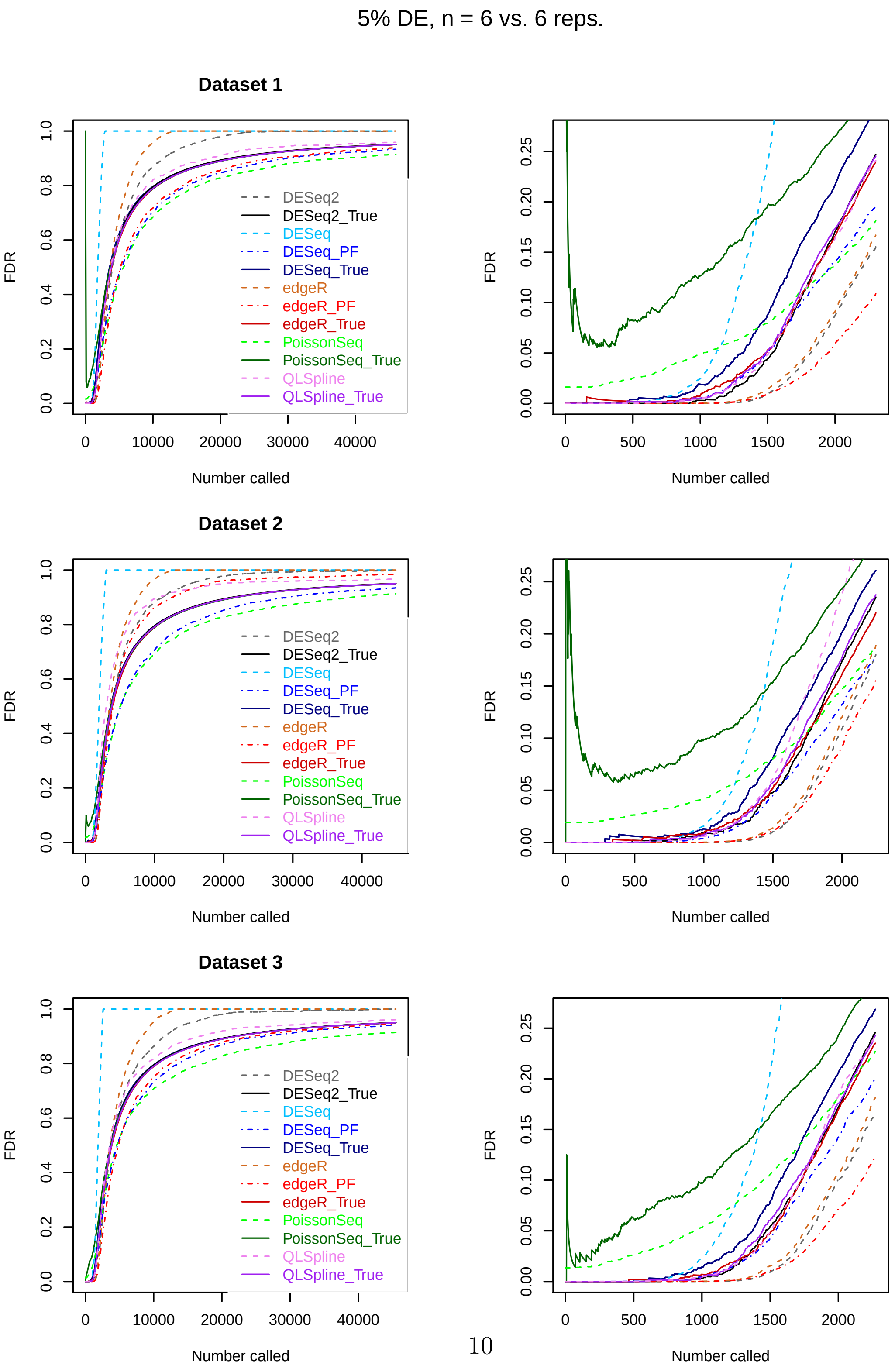

Figure S9: $n = 6$, DE $= 5\%$

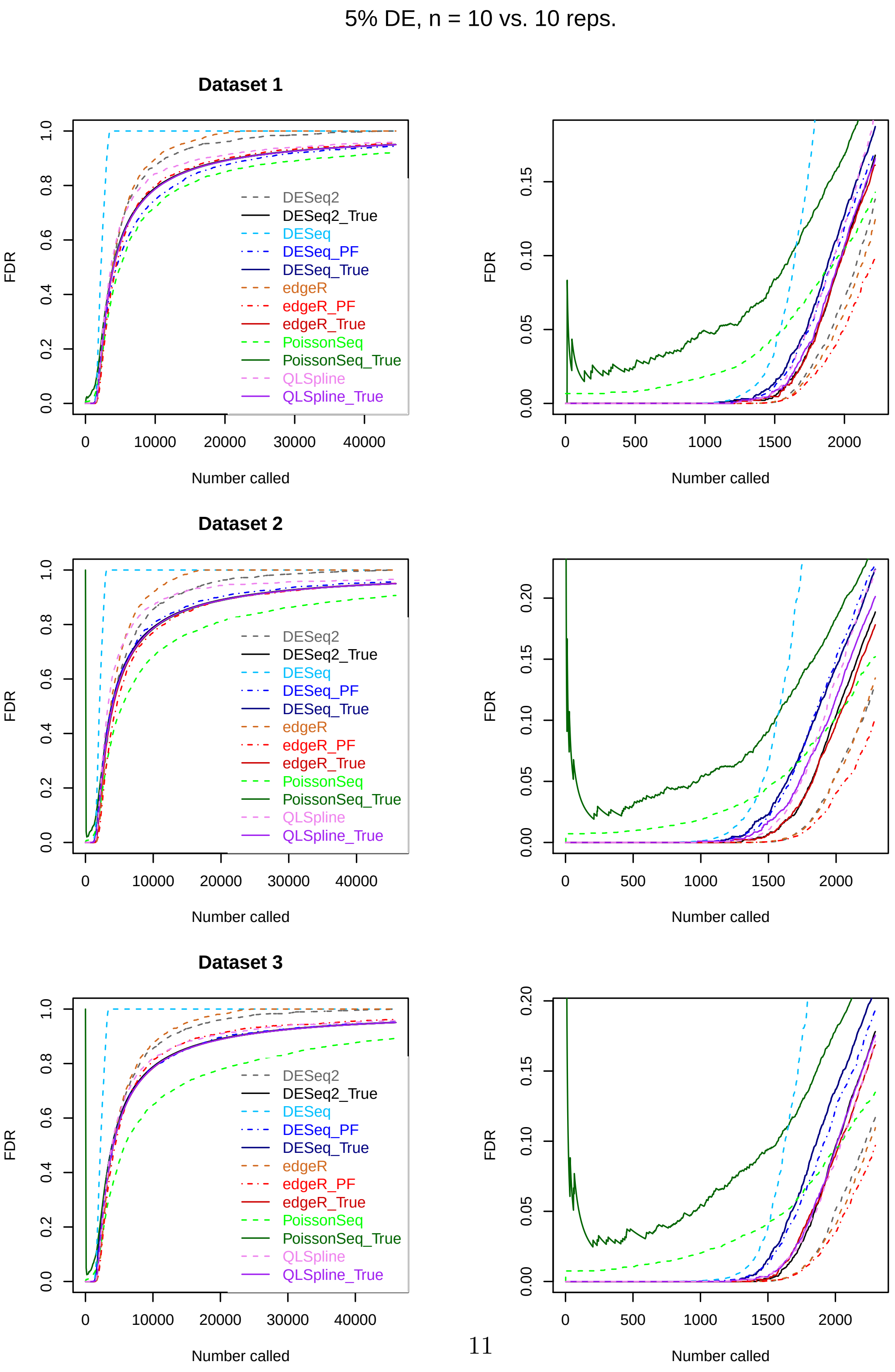

Figure S10: $n = 10$, DE $= 5\%$

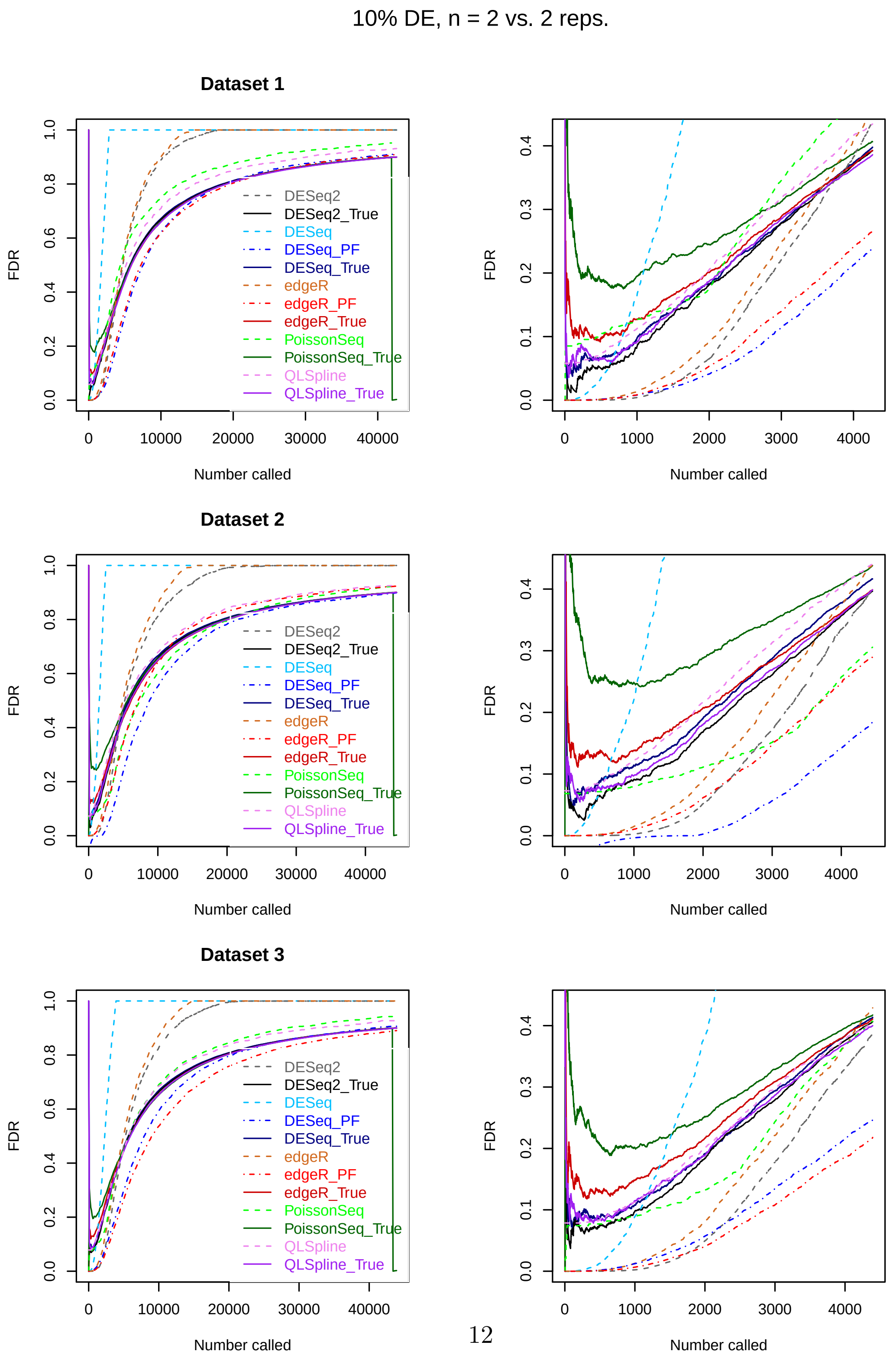

Figure S11: $n = 2$, DE $= 10\%$

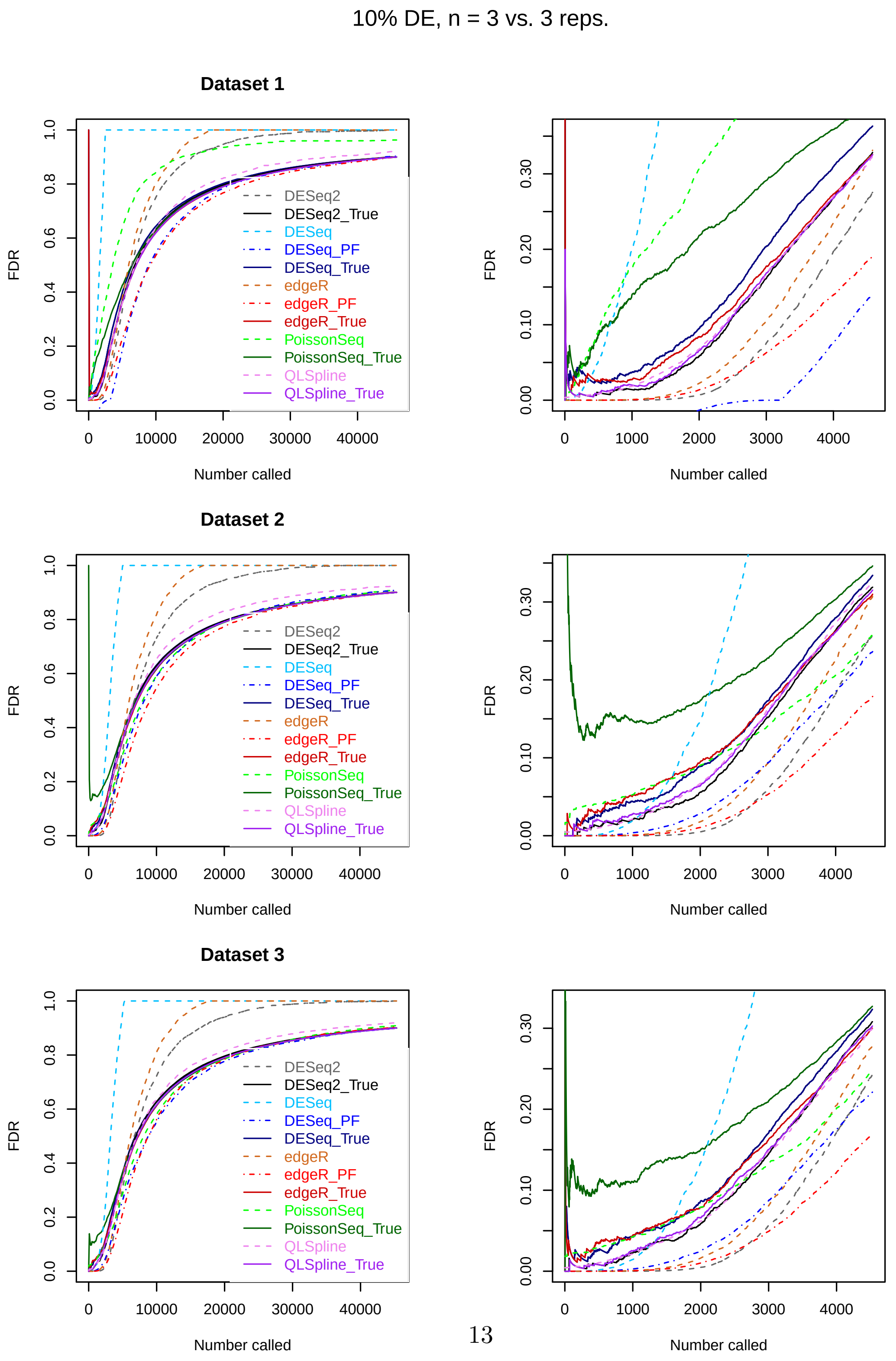

Figure S12: $n = 3$, DE $= 10\%$

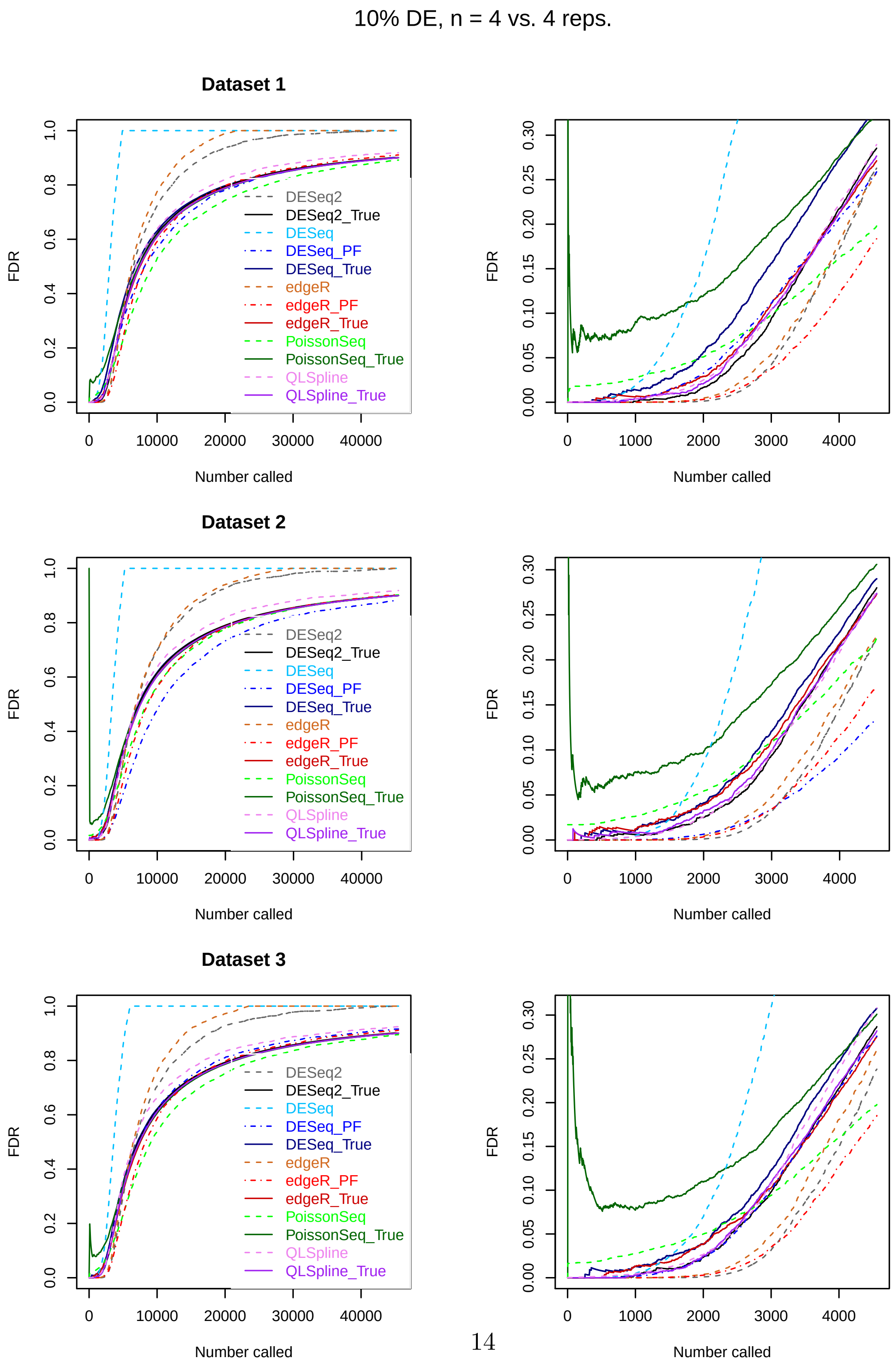

Figure S13: $n = 4$, DE $= 10\%$

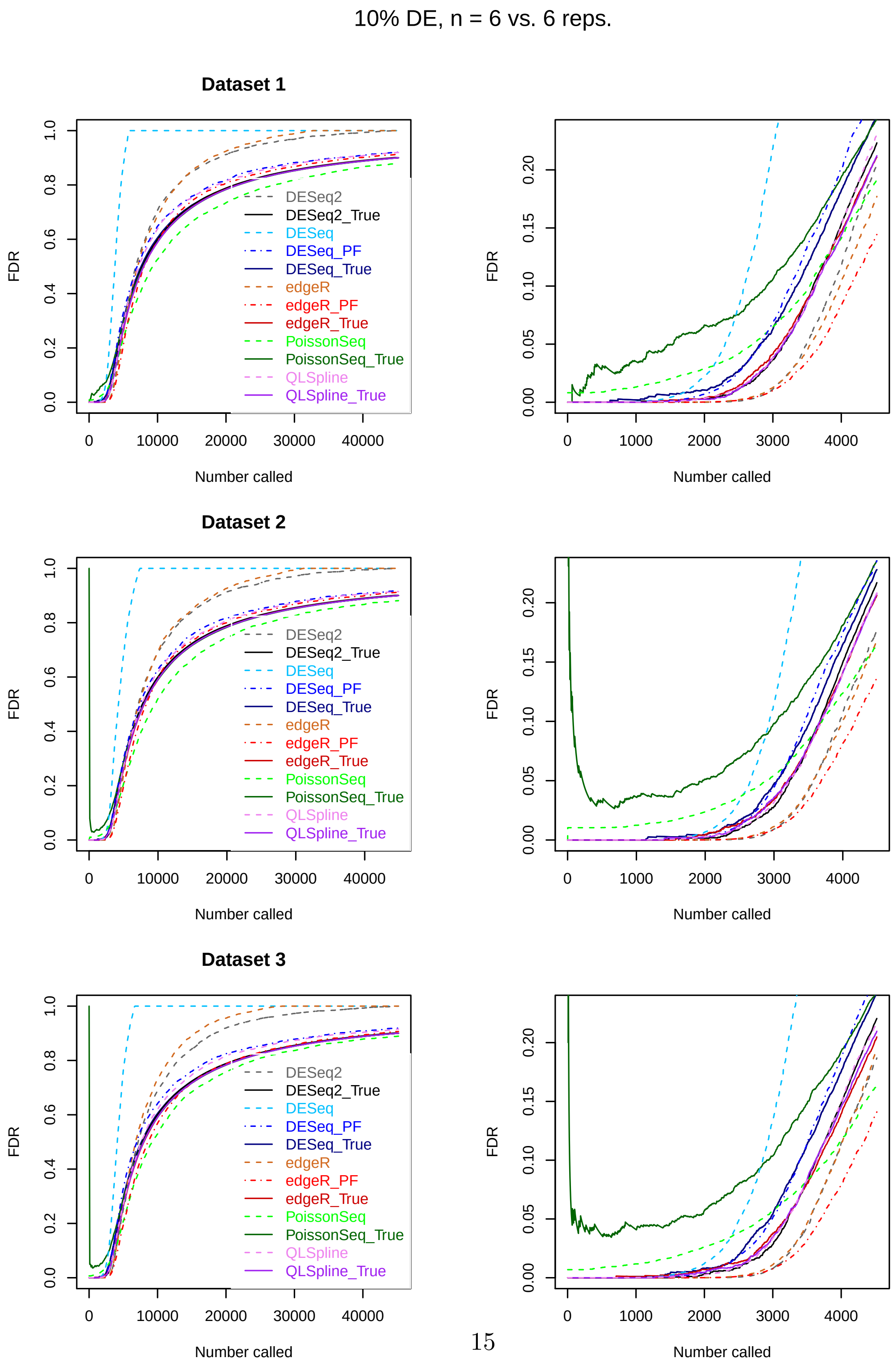

Figure S14: $n = 6$, DE $= 10\%$

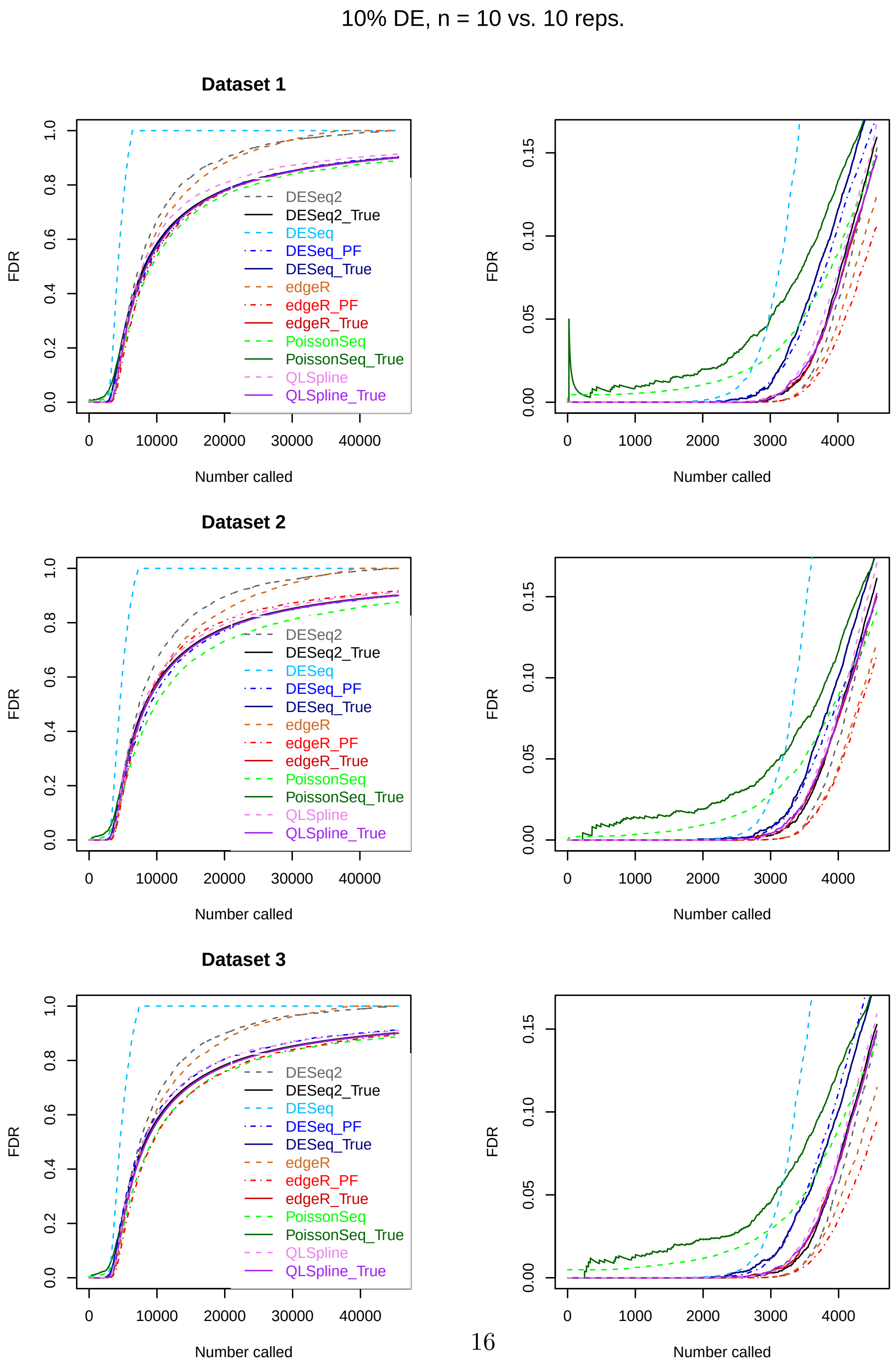

Figure S15: $n = 10$, DE $= 10\%$

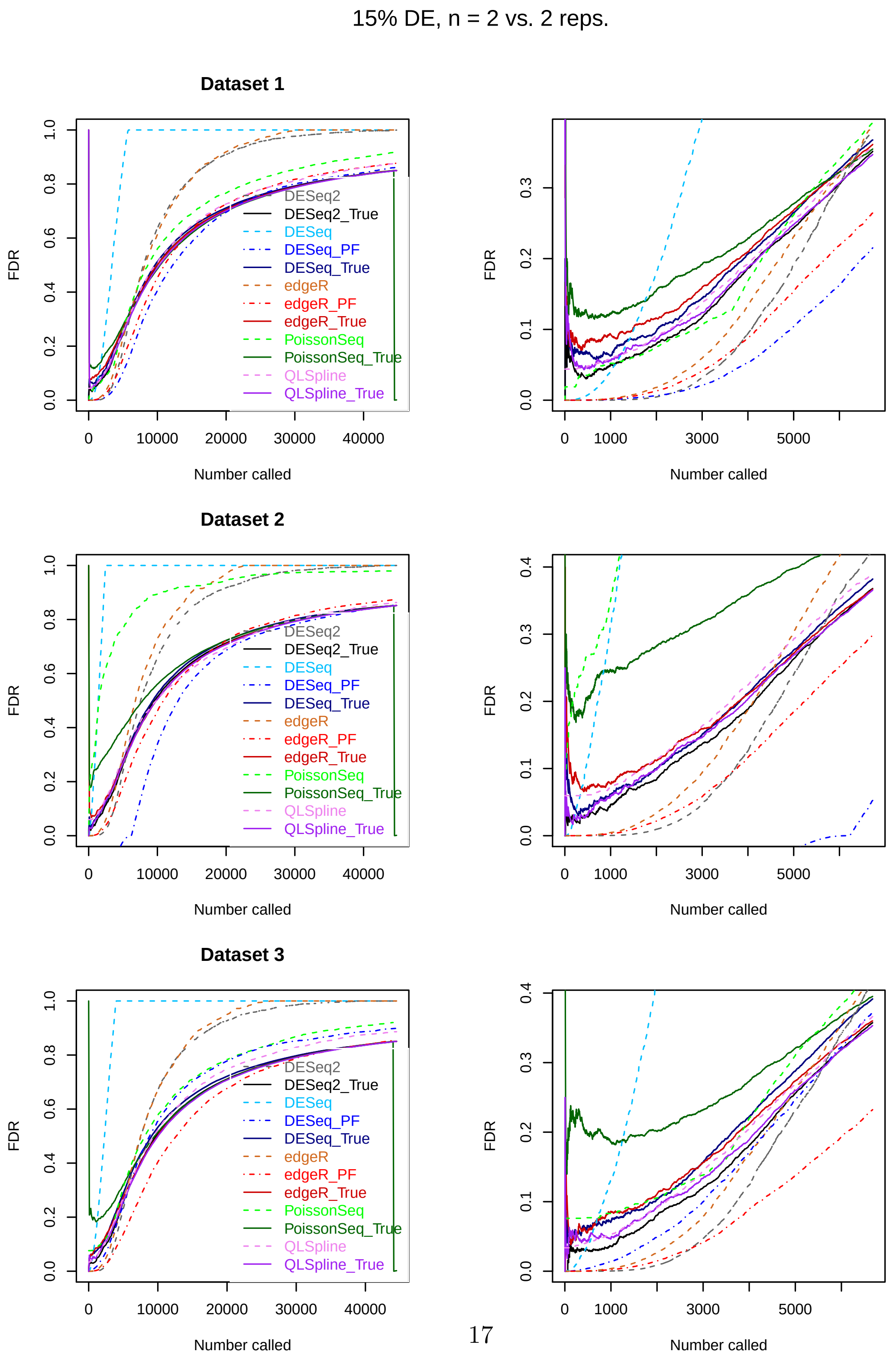

Figure S16: $n = 2$, DE $= 15\%$

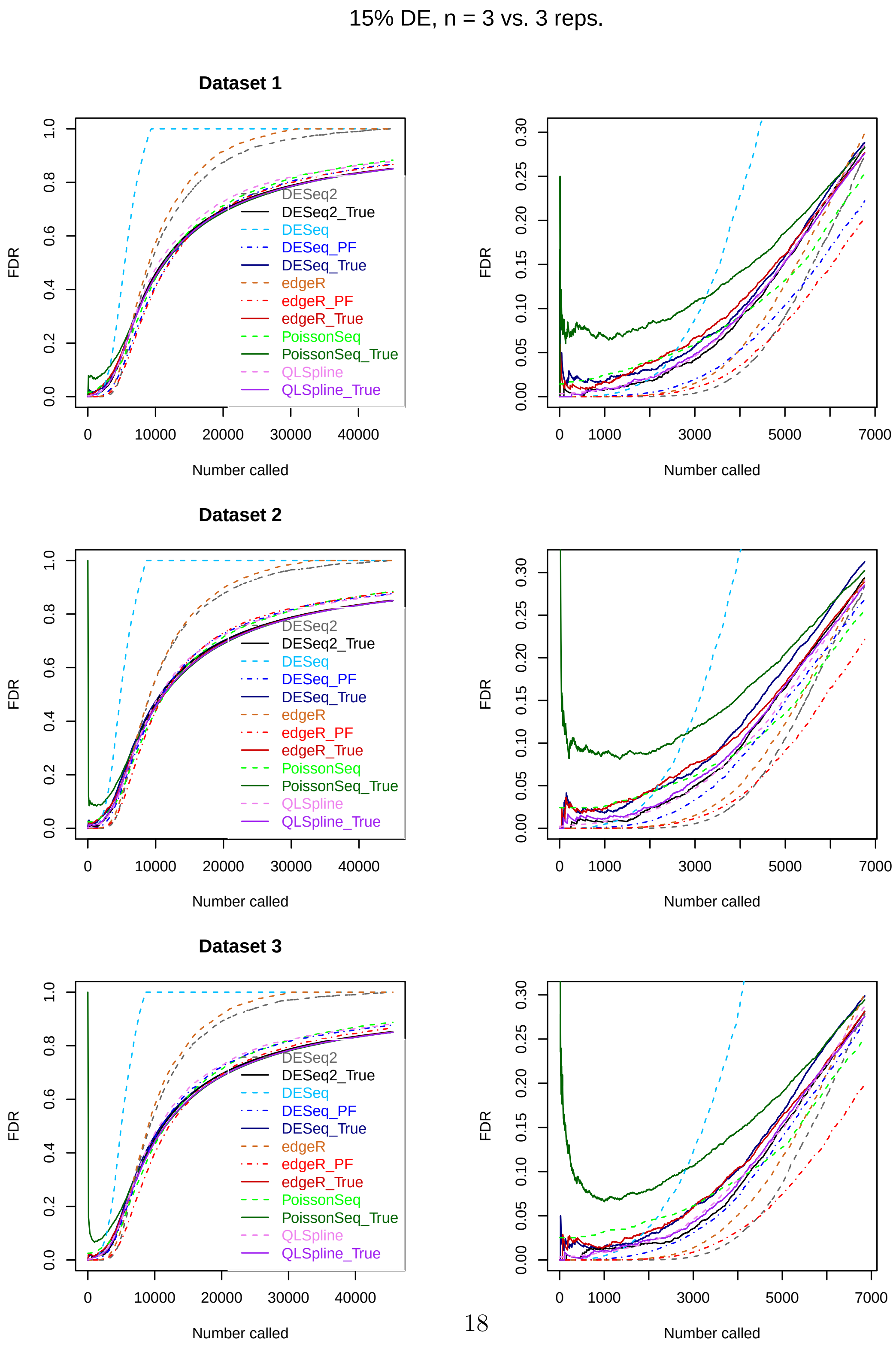

Figure S17: $n = 3$, DE $= 15\%$

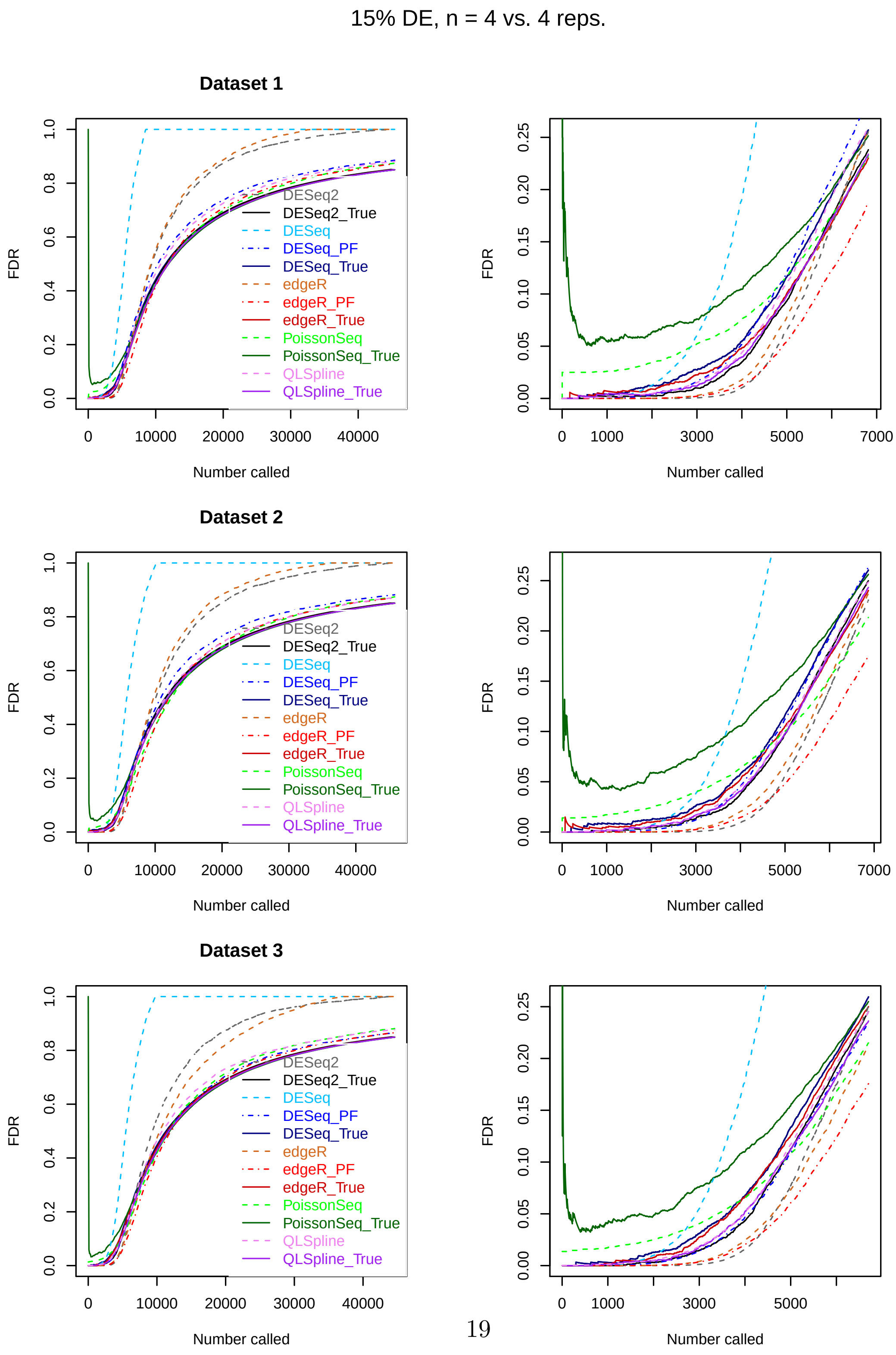

Figure S18: $n = 4$, DE $= 15\%$

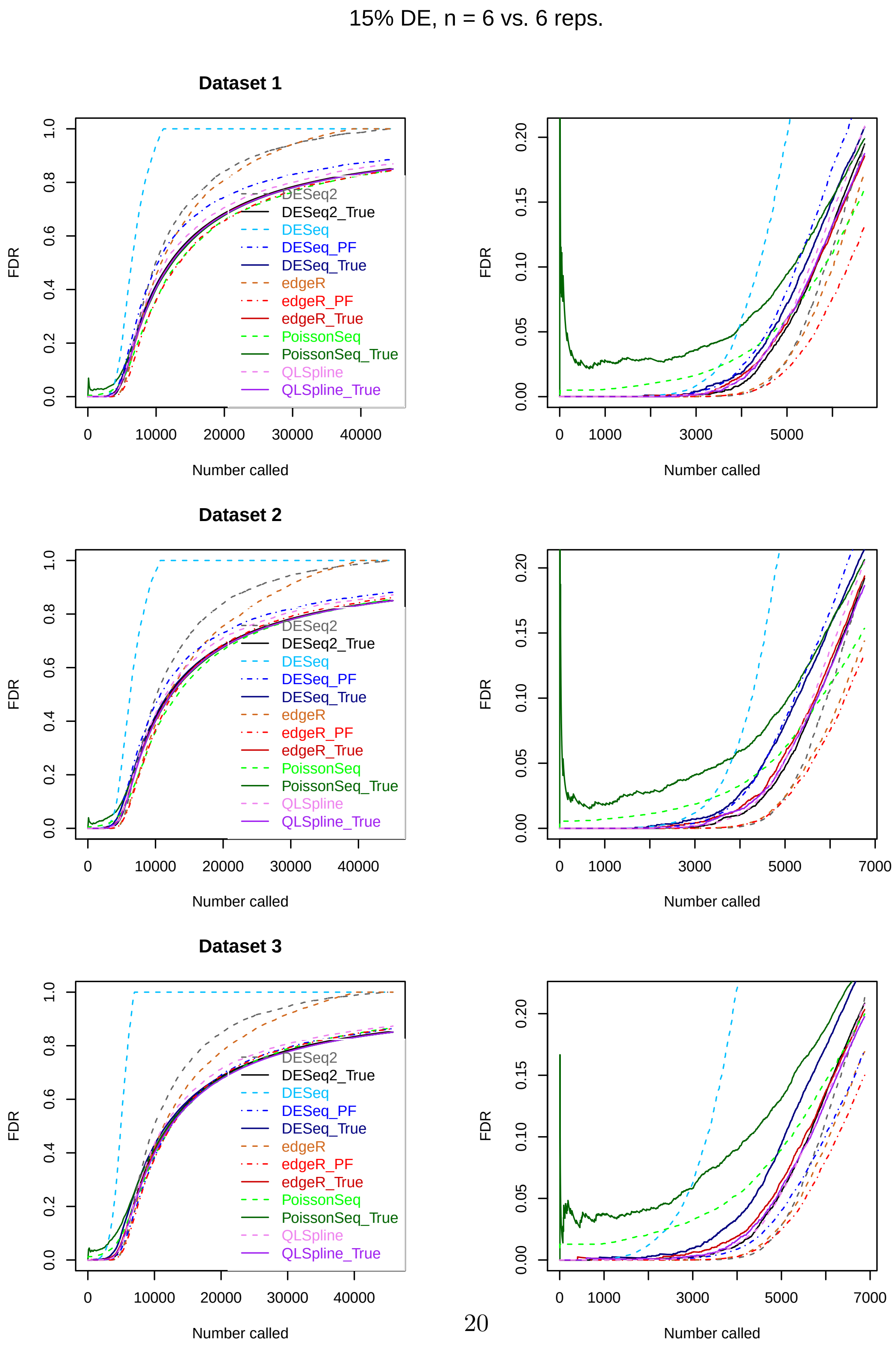

Figure S19: $n = 6$, DE $= 15\%$

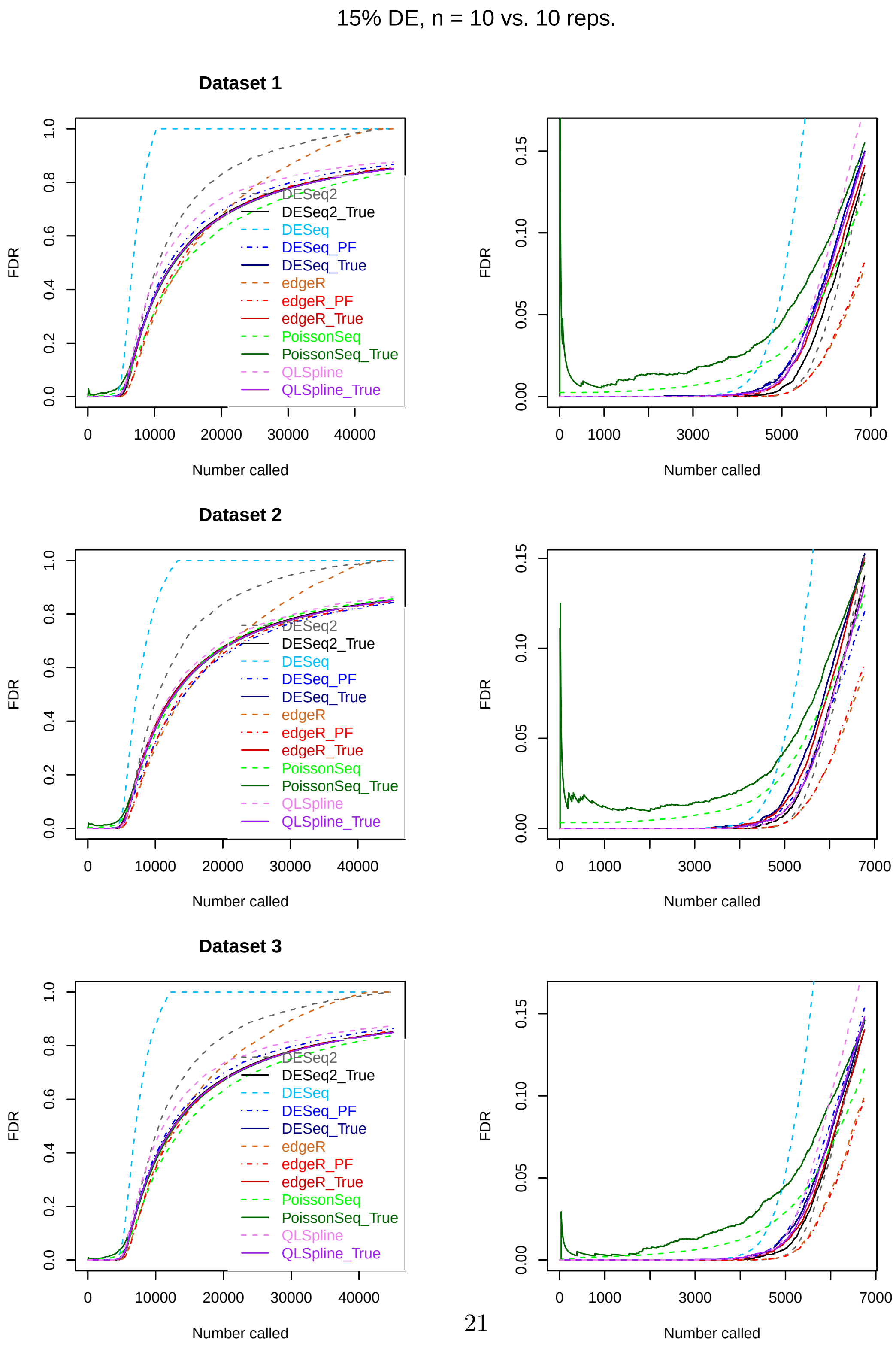

Figure S20: $n = 10$, DE $= 15\%$

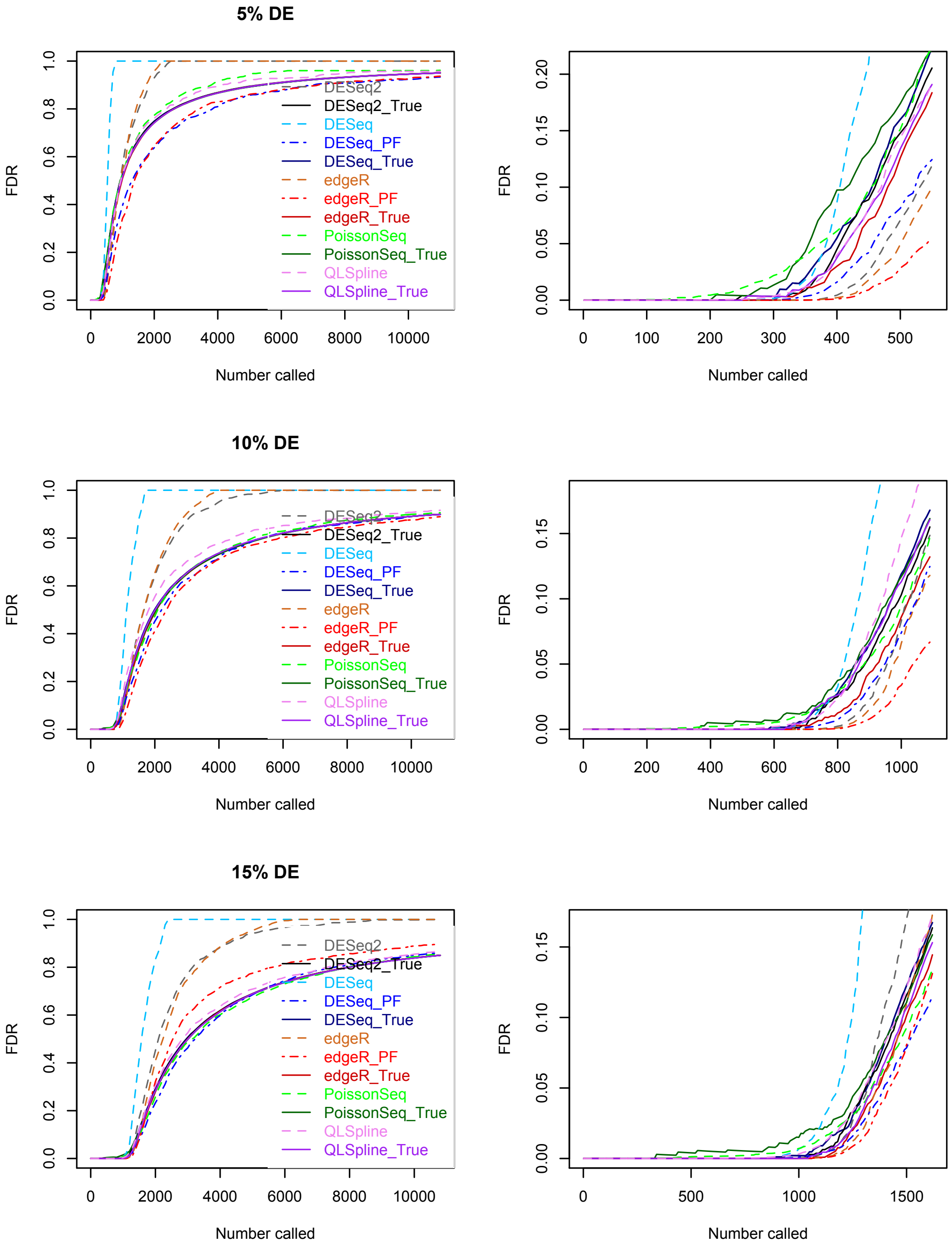

Figure S21: $n = 4$ replicates, DE $= 5, 10$ and 15%, mean and overdispersion parameters estimated from Bottomly data.

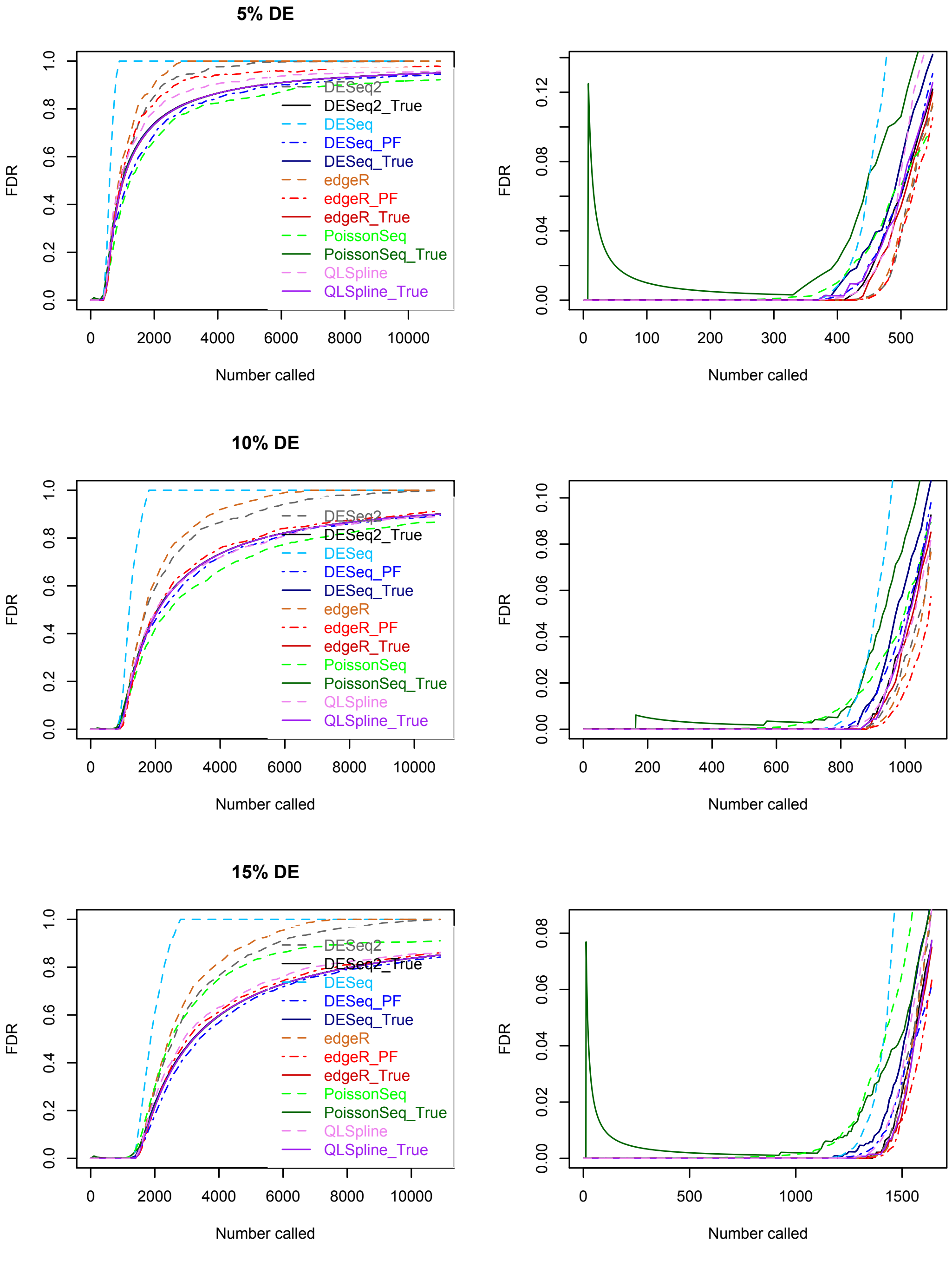

Figure S22: $n = 10$ replicates, DE $= 5, 10$ and 15%, mean and overdispersion parameters estimated from Bottomly data. The spikes at the lower end of the right-hand plots are an artifact of a single false discovery by Poisson-seq.

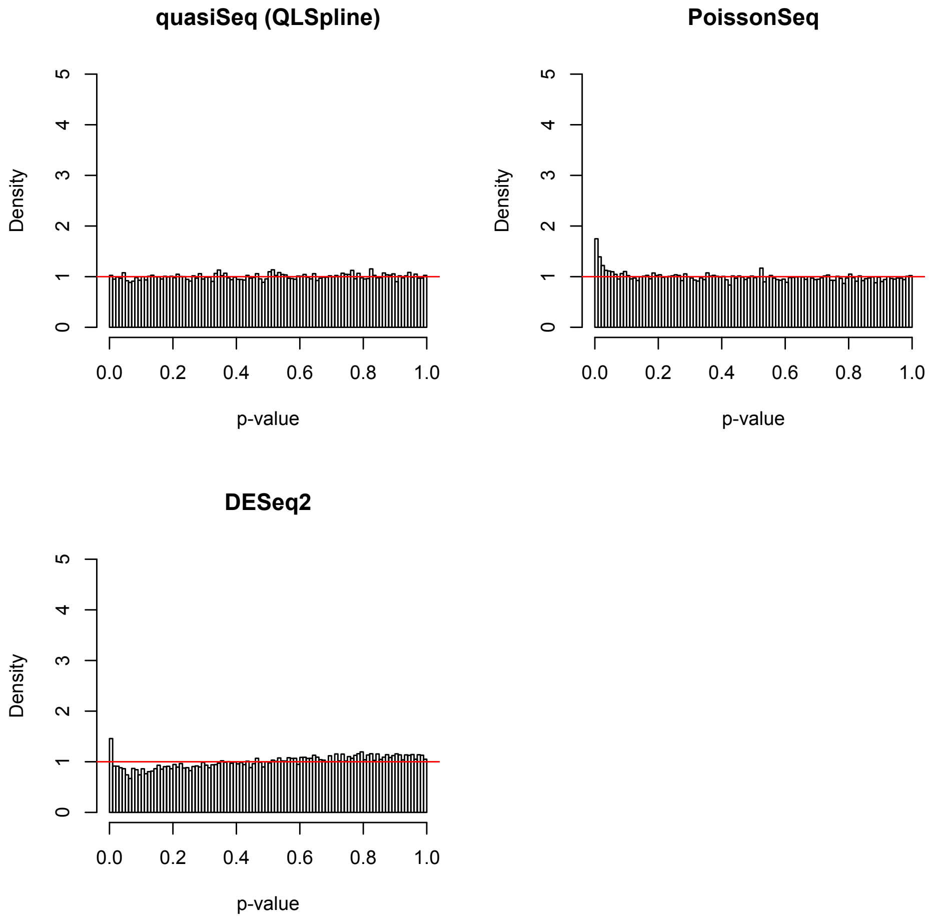

Figure S23: Distribution of p-values produced by the packages QLSpline, PoissonSeq and DESeq2 for $n = 4$ replicates of synthetic data, with no genes DE. For these packages, no 'flagpole' occurs in the histogram as p-values are calculated for test statistics with continuous distributions. The red line is the ideal uniform distribution expected when no genes are DE.

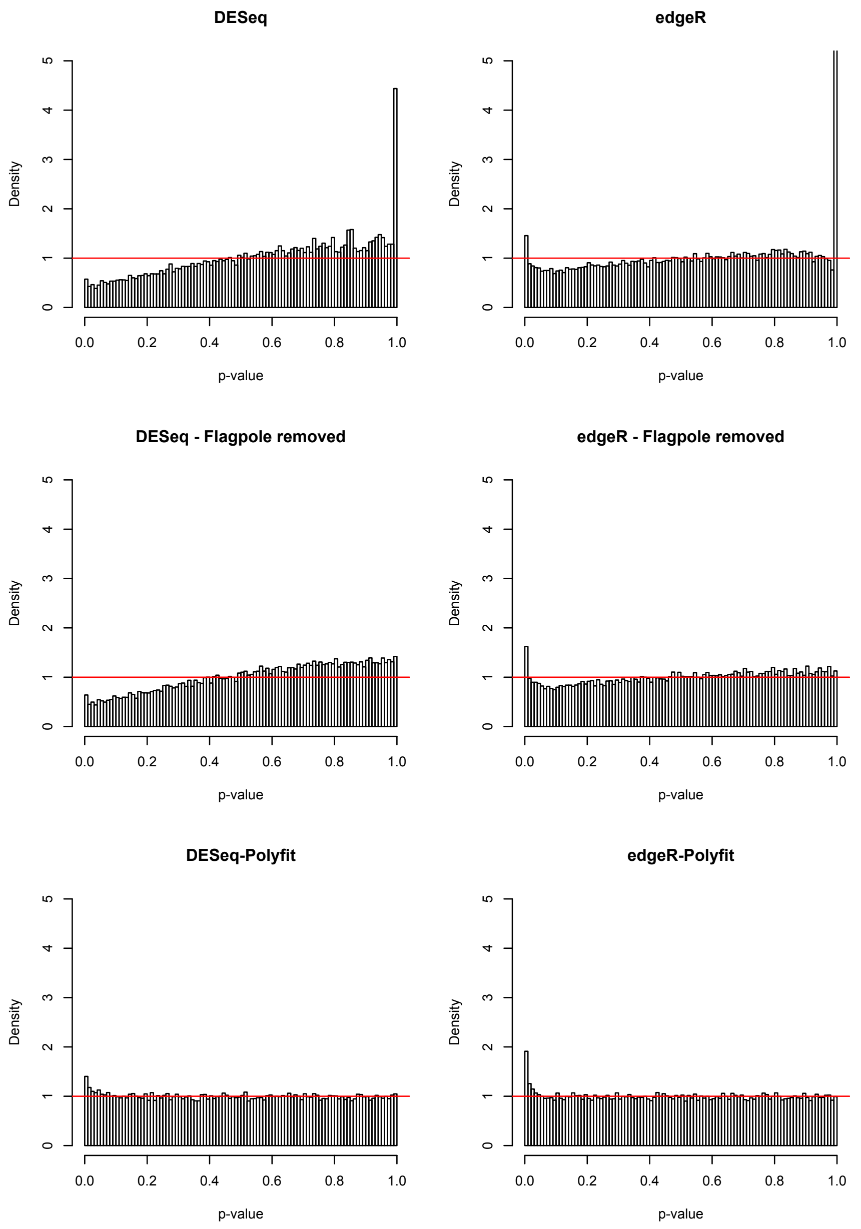

Figure S24: Distribution of p-values produced by the packages DESeq and edgeR for $n = 4$ replicates of synthetic data, with no genes DE (top row). Both packages typically produce upward-sloping histograms for non-DE genes with a 'flagpole' at $p = 1$. Also shown are histograms from the same data after removal of histograms using the first step of the Polyfit procedure, flagpole removan (middle row) and the second step, the adapted Storey-Tibshirani procedure (bottom row). The red line is ideal the uniform distribution expected when no genes are DE.

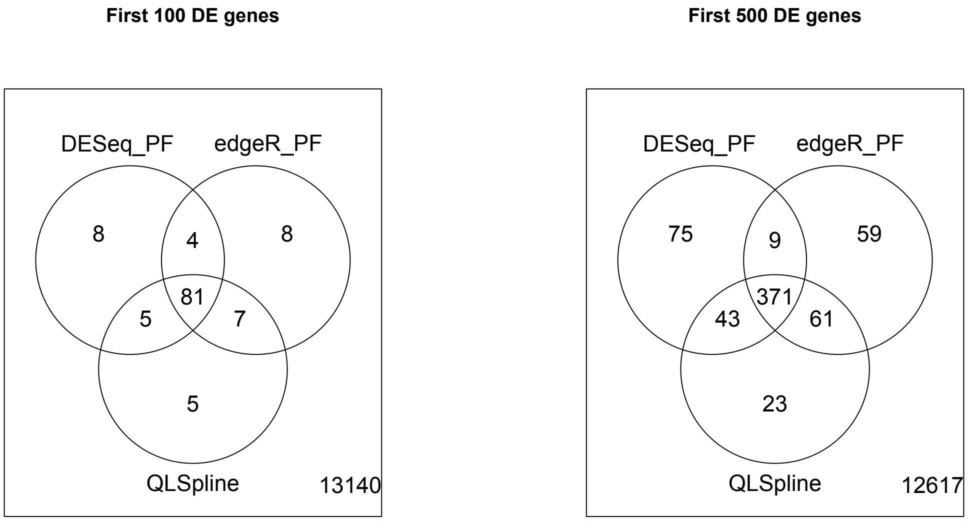

Figure S25: Venn diagrams of the most significant 100 and 500 genes called differentially expressed out of a total of 13,258 genes by QLSpline, Polyfit-DESeq and Polyfit-edgeR for the fly dataset [3] consisting of $n = 2$ vs. 2 biological replicates of fly-embryo RNA-seq data.

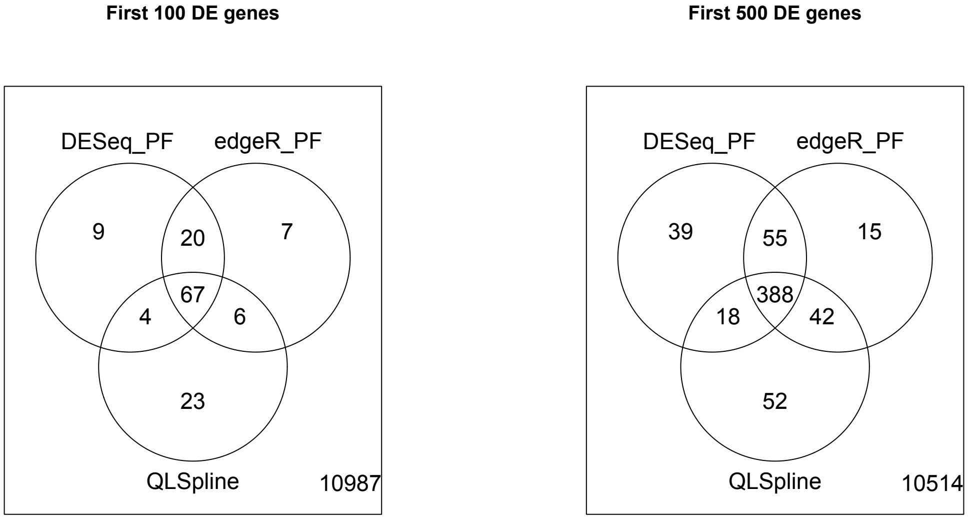

Figure S26: Venn diagrams of the most significant 100 and 500 genes called differentially expressed by QLSpline, Polyfit-DESeq and Polyfit-edgeR out of a total of 11,123 genes for the Bottomly dataset [5] consisting of $n = 10$ vs. 11 biological replicates of adult mouse brain RNA-seq data.

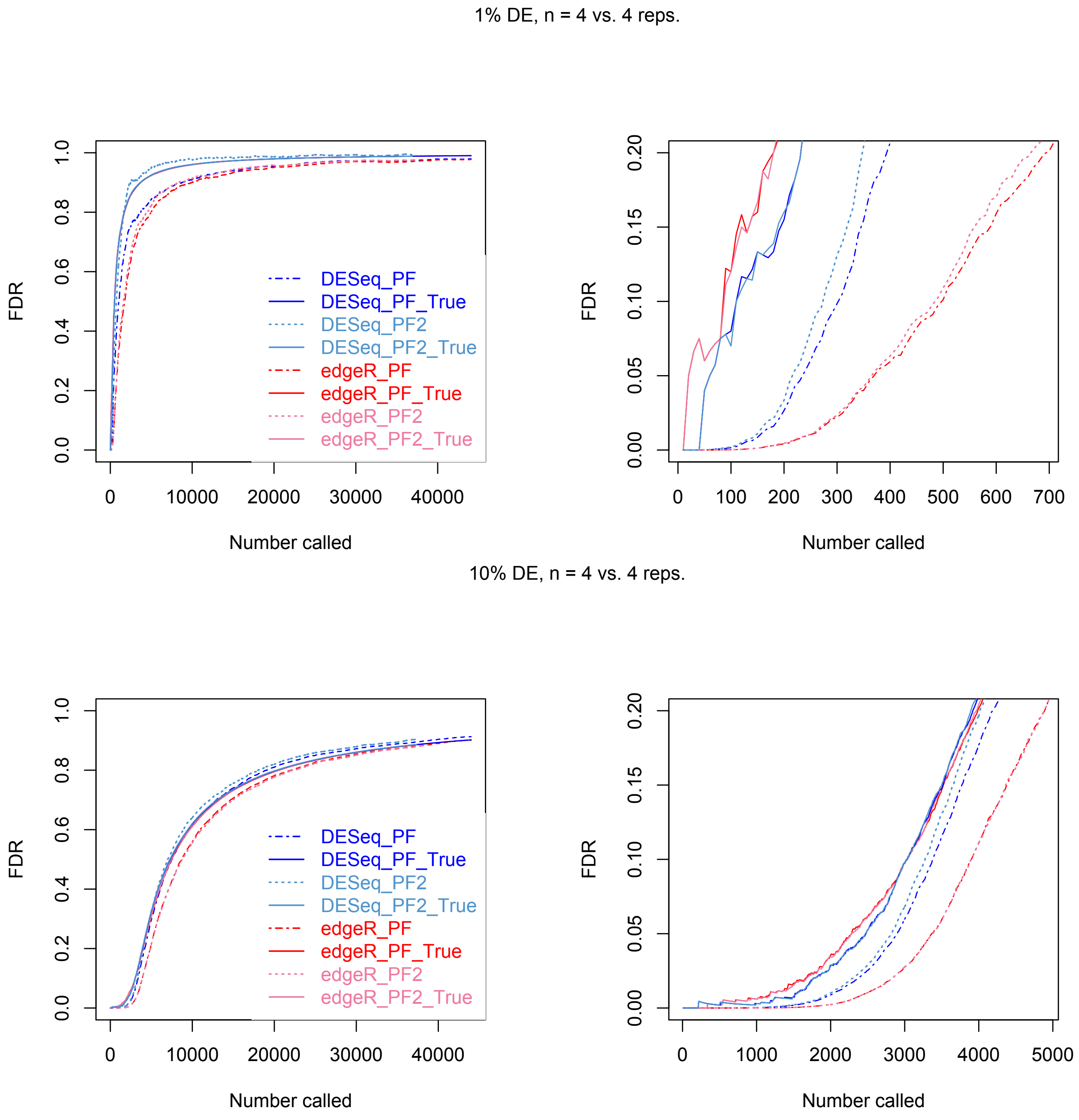

Figure S27: Comparison of FDR curves using the Polyfit procedure with 'flagpole removal described in Sections 4.1 and 4.2 (labelled PF) and the variant of the Polyfit procedure described in Section 4.3 (labelled PF2) which fits the p-value spectrum to a polynomial over the interval $[\lambda, 0.9]$.
